# Pair-bonding and social experience modulate new neurons survival in adult male and female prairie voles (*Microtus ochrogaster*)

**DOI:** 10.3389/fnana.2022.987229

**Published:** 2022-09-15

**Authors:** Analía E. Castro, Raymundo Domínguez-Ordoñez, Larry J. Young, Francisco J. Camacho, Daniela Ávila-González, Raúl G. Paredes, Nestor F. Díaz, Wendy Portillo

**Affiliations:** ^1^Instituto de Neurobiología, Universidad Nacional Autónoma de México (UNAM), Querétaro, Mexico; ^2^Benemérita Universidad Autónoma de Puebla, Complejo Regional Centro, Puebla, Mexico; ^3^Silvio O. Conte Center for Oxytocin and Social Cognition, Center for Translational Social Neuroscience, Department of Psychiatry and Behavioral Sciences, Emory National Primate Research Center, Emory University, Atlanta, GA, United States; ^4^Escuela Nacional de Estudios Superiores, Unidad Juriquilla, Universidad Nacional Autónoma de México (UNAM), Mexico City, Mexico; ^5^Instituto Nacional de Perinatología Isidro Espinosa de los Reyes, Mexico City, Mexico

**Keywords:** adult neurogenesis, pair bonding, olfactory bulbs, dentate gyrus of the hippocampus, social experience

## Abstract

Prairie voles are a socially monogamous species that, after cohabitation with mating, form enduring pair bonds. The plastic mechanisms involved in this social behavior are not well-understood. Neurogenesis in adult rodents is a plastic neural process induced in specific brain areas like the olfactory bulbs (OB) and dentate gyrus (DG) of the hippocampus. However, it is unknown how cell survival is modulated by social or sexual experience in prairie voles. This study aimed to evaluate if cohabitation with mating and/or social exposure to a vole of the opposite sex increased the survival of the new cells in the main and accessory OB and DG. To identify the new cells and evaluate their survival, voles were injected with the DNA synthesis marker 5-bromo-2’-deoxyuridine (BrdU) and were randomly distributed into one of the following groups: (A) Control (C), voles that did not receive any sexual stimulation and were placed alone during the behavioral test. (B) Social exposure (SE), voles were individually placed in a cage equally divided into two compartments by an acrylic screen with small holes. One male and one female were placed in opposite compartments. (C) Social cohabitation with mating (SCM), animals mated freely. Our findings demonstrated that SCM females had increases in the number of new cells (BrdU-positive cells) in the main olfactory bulb and new mature neurons (BrdU/NeuN-positive cells) in the glomerular layer (GlL). In contrast, these new cells decrease in males in the SE and SCM conditions. In the granular cell layer (GrL), SCM females had more new cells and neurons than the SE group. In the accessory olfactory bulb, in the anterior GlL, SCM decreased the number of new cells and neurons in females. On the other hand, in the DG, SCM and SE increase the number of new cells in the suprapyramidal blade in female voles. Males from SCM express more new cells and neurons in the infrapyramidal blade compared with SE group. Comparison between male and females showed that new cells/neurons survival was sex dependent. These results suggest that social interaction and sexual behavior modulate cell survival and influence the neuronal fate in a sex-dependent manner, in the OB and DG. This study will contribute to understand neural mechanisms of complex social and pair bond behaviors in the prairie voles; supporting adult neurogenesis as a plastic mechanism potentially involved in social monogamous strategy.

## Introduction

*Microtus ochrogaster* (prairie voles) are monogamous rodents that develop life-long pair bonds, showing a highly social organization and displaying biparental care for their offspring. Social behaviors associated with pair bonding involve multiple neurobiological processes including those regulated by region-specific expression of oxytocin and vasopressin receptors (Walum and Larry, 2018; Froemke and Young, 2021; Han et al., 2021; Inoue et al., 2022; Rigney et al., 2022) and plastic neural processes such as neurogenesis (review in Holmes, 2016; Bedos et al., 2018). Pair bonds are established after cohabitation without sex for 24 h or mating for 6 h (Williams et al., 1992). Thus, mating accelerates pair bonding.

In rodents, social behaviors and mating require the recognition of conspecifics through the olfactory system. The olfactory bulb (OB) is constituted by the main (MOB) and accessory olfactory bulb (AOB). The first receives odor information from the olfactory epithelium, whereas the second from the vomeronasal organ. In addition, the AOB is divided anatomically and functionally into two sub-regions, anterior (aAOB) and posterior (pAOB), characterized by different physiological functions and connections (Mandairon and Linster, 2009). The aAOB is involved in processing reproductive olfactory cues. In contrast, the pAOB participates in modulating defensive and aggressive behaviors and is activated in response to sexual volatile odors and male major urinary proteins (Brennan et al., 1999; Kumar et al., 1999; McGregor et al., 2004; Yoshikage et al., 2007; Huilgol et al., 2013).

Olfactory circuits play a fundamental role in sexual and socio-sexual behaviors in prairie voles. Indeed, bilateral olfactory bulbectomy affects social and affiliative responses in both sexes. Notably, sexual, paternal, and social behaviors are reduced in male voles after OB removal (Kirkpatrick et al., 1994). In females, OB lesion eliminated partner preference and decreases estrus induction following exposure to males (Williams et al., 1992). On the other hand, vomeronasal organ injury provokes a decrease in the reproductive efficiency of male voles after prolonged cohabitation with a sexually receptive female (Lepri and Wysocki, 1987) and impairs mating-induced pair bonding in females (Curtis et al., 2001).

The OB is connected with the hippocampus *via* the entorhinal cortex and perforant path (Vanderwolf, 1992). The entorhinal cortex sends information to the DG, the input region of the hippocampus, considered as a preprocessor of the incoming information to CA3 (Amaral and Witter, 1989; Jonas and Lisman, 2014); whereas CA1 projects to the anterior olfactory nucleus and olfactory bulb (van Groen and Wyss, 1990). The OB and the hippocampus are essential components of the limbic system which regulates social and sexual input such as conspecific odor signaling, partner recognition, spatial information, and partner-associated memory in rodents (Catani et al., 2013). The DG of the hippocampus is critical for social memory and is proposed to play a critical role in pair bonding in titi monkeys and prairie voles (Tabbaa et al., 2016; Walum and Larry, 2018; Baxter et al., 2020) and expresses high amounts of oxytocin receptors (Inoue et al., 2022). Furthermore, oxytocin in the anterior DG and CA2/CA3 is necessary for discrimination of social stimulus (Raam et al., 2017). Interestingly, these neuronal regions modify their activity and functional organization in response to environmental and physiological stimulation. It is well-recognized that adult neurogenesis is a mechanism of brain plasticity and has been implicated in sexual and parental behavior, mate selection, recognition of sexual partner, memory, and learning, etc. Conversely, these behaviors promoted adult neurogenesis (review in Holmes, 2016; Bedos et al., 2018).

Previous studies demonstrated that sexual behavior modulates adult OB and DG neurogenesis in rodents. For example, in male mice, the first mating experience increases the percentage of new neurons in the glomerular layer (GlL) of the MOB (Velazco-Mendoza et al., 2019), and sexual stimulation originates new neurons in the AOB in male rats (Unda et al., 2016). Also, paced mating increases the number of new neurons in the granular layer (GrL) of the AOB, and repeated sexual stimulation increases the percentage of new neuronal cells in the GrL of the MOB in female rats (Arzate et al., 2013; Alvarado-Martínez and Paredes, 2018; Portillo et al., 2019). Moreover, acute and chronic sexual experience (1 or 14 mating sessions) promotes neurogenesis and dendritic growth in different cell subpopulations of the DG in male rats (Leuner et al., 2010); whereas one or four mating sessions increases the new cells number that arrive to the DG in female rats (Portillo et al., 2012).

Socio-sexual behavior also modulates adult neurogenesis in prairie voles. In males, our research group demonstrated that social and sexual experiences increase the percentage of new neuroblasts in the dorsal subventricular zone (SVZ) Furthermore, we observed an increase in number of new neuroblasts in the anterior rostral migratory system (RMS) in male that had a sexual experience as compared with males socially exposed to a sexually receptive female (Castro et al., 2020). In addition, social and sexual stimulation increases the number of new immature cells in the subgranular zone of the DG of the hippocampus and hilus in male prairie voles. Cohabitation with mating and social exposure to another male increases immature neuronal fate in the infrapyramidal blade of the DG as compared with control animals (Castro et al., 2020). In female voles, sexual exposure to non-family-related male odors boosts cell proliferation in the RMS and favors the neuronal lineage commitment (Smith et al., 2001). These results suggest socio-sexual experiences can modulate the number of new cell/neurons born in the germinal niches (SVZ and subgranular zone of the DG) (Smith et al., 2001; Castro et al., 2020). Nevertheless, studies are needed to elucidate if these new generated cells/neurons populations arrive and survive in the OB and DG regions.

Interestingly, adult neurogenesis is sexually dimorphic in rodents (Peretto et al., 2001; Nunez-Parra et al., 2011; Glasper et al., 2018). In prairie voles, the number and diameter of the neurospheres derived from the ventricular zone and granular zone of the DG were higher in female as compared with males, suggesting that female neural stem cells had an enhanced proliferative potential under cell culture conditions (Ávila-González et al., 2020).

The present study aims to evaluate if social cohabitation with mating that induces pair bonding and/or social exposure to an opposite-sex conspecific could regulate the survival of new cells/neurons in the OB and DG of the hippocampus in adult female and male prairie voles. Based on the findings presented above, we hypothesize that in male and female prairie voles, social exposure and social cohabitation with mating will increases the number of new cells/neurons that survive in the granular and glomerular layer of the MOB and AOB and in the infrapyramidal blade of the DG. For sex comparisons, we predict that females would have more new cells/neurons than survive than males.

## Materials and methods

### Animal treatment

Adult male and female voles were obtained from our local colony established at the Instituto de Neurobiologia, UNAM. Animals were housed in a 14 h:10 h light:dark cycle (Williams et al., 1992; Kirkpatrick et al., 1994; Curtis et al., 2001; Lieberwirth et al., 2012; Zheng et al., 2013) and the lights were turn on at 08:00 h, with access to food (rabbit high-fiber diet 5326 LABDIET, oats, and sunflower seeds) and water *ad libitum*. All experimental protocols were performed in accordance with the “Reglamento de la Ley General de Salud en Materia de Investigacion para la Salud” of the Mexican Health Ministry, which is based on NIH guidelines for the use and care of animals in research. The protocols were approved by the Animal Care Committee of the Instituto de Neurobiologia (072) and Instituto Nacional de Perinatologia (2018-1-163).

### Experimental procedure

**FIGURE 1:**
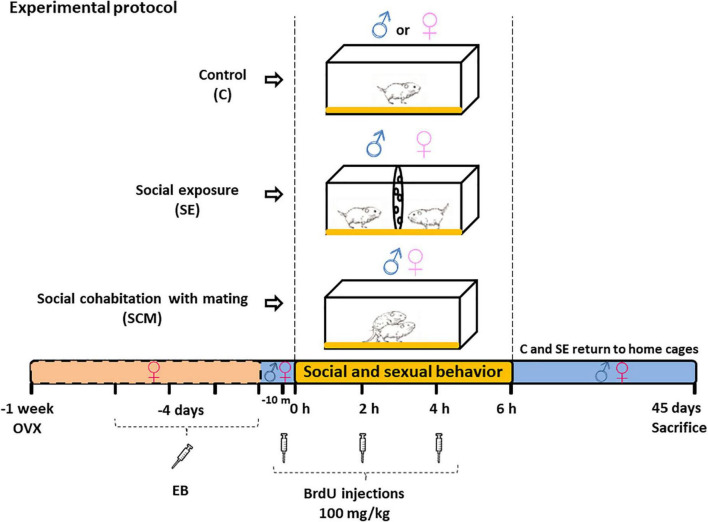
Diagram of the experimental protocol experimental conditions for Control (C), social exposure to a conspecific of the opposite sex (SE) and social cohabitation with mating (SCM) animals. BrdU (5-bromo-2-deoxyuridine) was injected 10 min before the behavioral test and 2 and 4 h after the test. EB, estradiol benzoate; OVX, bilateral ovariectomy.

Adult female voles were ovariectomized and sexual receptivity was induced by daily subcutaneous administration of estradiol benzoate (EB, 0.5 μg/vole, Sigma-Aldrich) for 4 consecutive days before the behavioral test (Smale et al., 1985; Roberts et al., 1998; Ulloa et al., 2018), whereas male voles were sexually intact. Males and females, 3–4 months old, came from different mating units housed in separate same-sex home cages. Experimental groups were designed according to our previous report (Castro et al., 2020); here, twenty male and twenty female voles were randomly distributed in three groups: (1) Control (C, *N* = 7): animals were placed alone in a clean cage; (2) social exposure (SE, *N* = 6): male and receptive female voles were placed in a clean plastic cage with two compartments separated by a plastic screen with small holes, the female was place in one compartment and in the opposite one the male, where both could receive sensory stimulation but were not allowed physical contact; (3) social cohabitation with mating (SCM, *N* = 7): male and female voles were able to mate, which led to pair bonding. The behavioral test lasted 6 h. To label new cells, three i.p. injections of DNA synthesis marker 5-bromo-2′-deoxyuridine (BrdU; 100 mg/Kg) were administered, 10 min before the beginning of the test, 2 and 4 h later. After 6 h, animals from C and SE groups were returned to home cages with their sibling, whereas SCM couples stayed together until sacrificed (Figure 1). This procedure was necessary since voles establish a pair bond after 24 h of cohabitation without mating (Williams et al., 1992). Thus, our paradigm allowed us to differentiate the pair bond induced by social cohabitation with mating from that induced by long cohabitation periods without mating. Additionally, for the SCM the protocol enables label neurons newly born during the social cohabitation with mating but does not determine whether it is the mating or the 45 days of cohabitation that effects survival of the labeled neurons.

Forty-five days later, voles were anesthetized with an overdose of i.p. pentobarbital (6.3 mg/vole, Cheminova) and perfused intracardially with 0.1 M phosphate-buffered saline (PBS) followed by 4% paraformaldehyde-PBS (PFA, Sigma-Aldrich). Vole brains were removed, post-fixed by immersion in 4% PFA-PBS for 1 h and stored in 30% sucrose (J.T. Baker)-PBS solution at 4°C.

### Immunofluorescence assay

Male and female brains were cut into thirty-micrometer slices and stored in a cryoprotective solution until immunostaining assay. Three brain sections per animal per area containing the OB (MOB and AOB; sagittal plane) and the DG of the hippocampus (coronal plane) were used.

Free-floating brain slices were washed three times for 5 min each in Tris-buffered saline solution, TBS pH 7.6. Then, sections were incubated in 1% H_2_O_2_-TBS solution for 30 min at room temperature (RT) and washed three times for 5 min each in TBS. Subsequently, slices were incubated in 2N HCl 37^°^C for 30 min and washed three times for 5 min in TBS. To enhance BrdU signal and NeuN labeling, a combination of tyramide signal amplification (TSA) and an ABC system were used (TSA Plus Cyanine 3 System NEL744001KT, TSA Plus Coumarin System NEL703001KT PerkinElmer Inc. and Vectastain ABC kit PK-6100, Vector Laboratories, respectively). The slices were incubated in blocking buffer (0.5% w/v bovine serum albumin fraction V A9418 (Sigma-Aldrich) diluted in TBS) for 30 min at RT rinsed and incubated overnight in rat anti-BrdU (OBT0030 Serotec), dilution 1:800 in 0.05% blocking buffer, at 4^°^C. Then, they were rinsed three times for 5 min each in wash buffer, 0.1% TX-TBS, and incubated in anti-rat biotinylated antibody (BA-9400, VECTOR laboratories), diluted 1:2000 in 0.05% blocking buffer for 2 h at RT. Brain slices were incubated in AB reagent (25 μl from A and B solutions diluted in 5 ml TBS) for 1 h at RT and then washed three times for 5 min each in washing buffer. Brain sections were incubated with Cy3-Tyramide complex, diluted 1:100 in the kit-specific diluent for 10 min in a wet chamber and washed three times for 5 min each with washing buffer. A second overnight incubation with the primary biotinylated antibody mouse anti-NeuN 1:1,000 in 0.05% blocking buffer at 4^°^C was performed, followed by AB incubation for 1 h at RT. Slides were washed and then rinsed in the Coumarin-Tyramide complex (PerkinElmer Inc.) dilution 1:100 in the kit-specific diluent for 10 min RT in a wet chamber. Sections were washed, mounted, dried, covered with Aqua-Poly/Mount (Polysciences) and stored at 4^°^C in darkness until image processing.

### Cell counting

Brain slices were processed for double immunostaining using anti-BrdU and anti-NeuN primary antibodies. Photomicrographs were taken in a confocal microscope (Zeiss AX10) with a 20× objective (total magnification 200×) and digitalized with Mac Biophotonics ImageJ. Three slides per area per animal were quantified and each one was scanned into 4–6 planes in the z-axis. Cell count was performed in the central stack containing the better fluorescent labeling. Each photomicrograph was separated into the red and blue channels (BrdU and NeuN staining, respectively). To exclude unspecific particles, size (50-infinity) and circularity, filters (0.50–1.00) were applied using ImageJ’s particle analysis tool. Specific regions of interest (ROI) were drawn to delimit each cell layer in the OB and for the DG a freehand selection area was used. Each area was automatically calculated by the software. Finally, cell number/area was extrapolated to cell number/mm^2^.

**FIGURE 2:**
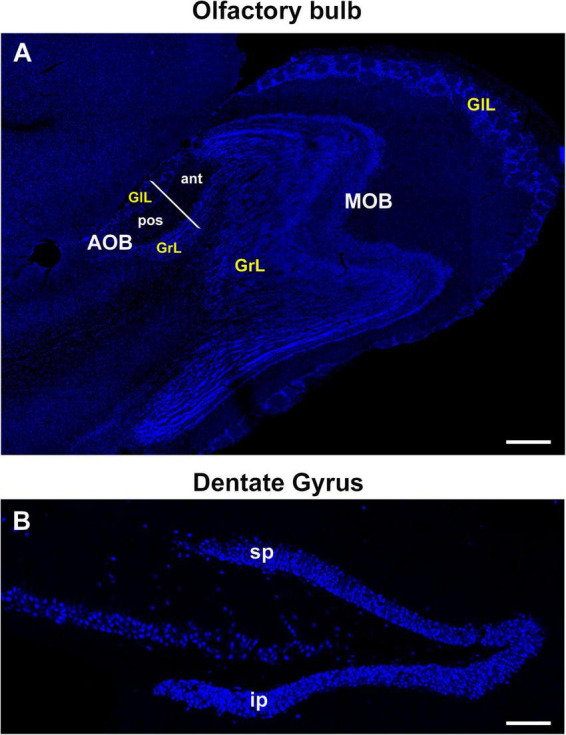
Representative sections of the olfactory bulb (sagittal plane) and dentate gyrus of the hippocampus (coronal plane) from adult vole brains stained with the nuclear marker Hoechst. **(A)** The glomerular and granular cell layers (GlL and GrL, respectively) of the main and accessory olfactory bulbs were analyzed (MOB and AOB, respectively). AOB was divided into the anterior and posterior zones. **(B)** Dentate gyrus of the hippocampus was divided into the supra and infrapyramidal blades (sp and ip, respectively) to perform cell counting analyses. 20×. Scale bar: 200 μm.

Double staining for BrdU/NeuN-positive cells was carried out in the MOB, AOB and DG (Figure 2). AOB was subdivided into the aAOB and pAOB and their GlL and GrL were analyzed, whereas positive cells were counted in the suprapyramidal and infrapyramidal blades of the DG, according to our preceding studies (Castro et al., 2020). The percentage of new neurons was calculated as the number of BrdU/NeuN-positive cells divided by the number of BrdU-positive cells and multiplied by 100.

The number of BrdU-positive cells was evaluated to determine if social and sexual stimuli increased the amount of new cells/neurons that arrive and survive in the examined areas. Moreover, the number and percentage of BrdU/NeuN-positive cells indicate whether the experimental conditions determine cell fate and survival into the neuronal linage.

### Data analysis

Data were analyzed using GraphPad Prism version 8.0.0 for Windows, San Diego, California, United States,^1^ and SigmaPlot 14.0 (Systat Software, Inc.). Female and male voles data were analyzed independently, since our data did not show a normal distribution, they were analyzed with a Kruskal-Wallis test followed by Dunn’s *post-hoc* test. Furthermore, a two-tailed Mann-Whitney U test was performed to analyze the differences between the data of male and female voles. A value of *p* < 0.05 was considered significant. Data are expressed as median plus interquartile ranges and the maximum and minimum values.

## Results

### Main olfactory bulb

#### Glomerular cell layer

**FIGURE 3:**
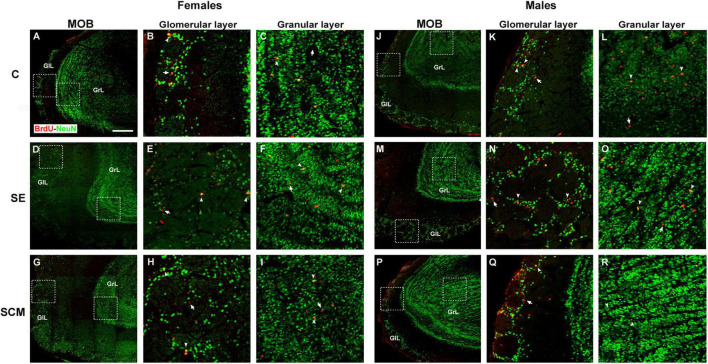
Representative photomicrographs of the new neurons that arrive and survive in the main olfactory bulb (MOB) in female and male adult prairie voles Photomicrographs of double immunolabeling for BrdU (red; proliferating cells) and NeuN (green; mature neurons) in the glomerular and granular cell layers: **(A–C)** Control (C); **(D–F)** social exposure (SE), and **(G–I)** social cohabitation with mating (SCM) females. **(J–L)** C; **(M–O)** SE and **(P–R)** SCM males. Scattered square: enlargement of the inset at 20×. White arrowheads: BrdU/NeuN-positive cells. White arrows: non-neuron BrdU-positive cells localized in the glomerular and granular cell layers (GlL and GrL, respectively). 20×. Scale bar: 100 μm.

**FIGURE 4:**
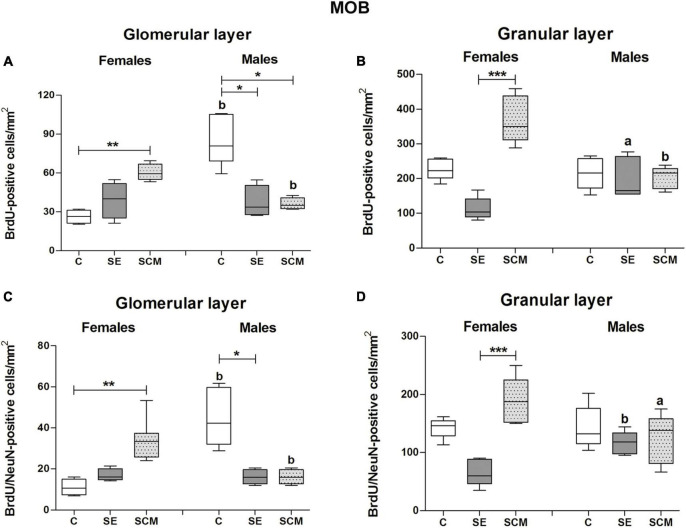
Number of BrdU and BrdU/NeuN-positive cells in the glomerular and granular cell layers of the main olfactory bulb (MOB) in control (C), Social exposure (SE), and Social cohabitation with mating (SCM) groups Kruskal-Wallis followed by Dunn’s *post-hoc* tests; *p < 0.05; **p < 0.01 and ***p < 0.001, were considered significant. Differences between female and male prairie voles were analyzed using the Mann-Whitney *U* test. ^a^*p* < 0.05, and ^b^*p* < 0.01. Animals were excluded from the analysis if three well-preserved sections were not obtained. Female subjects; C = 4, SE = 4, SCM = 7. Male subjects; C = 7, SE = 5, SCM = 5. ^a^ mean different from the opposite sex *p* < 0.05. ^b^ mean different from the opposite sex *p* < 0.01.

Representative photomicrographs of the BrdU- (red) and BrdU/NeuN-positive cells (yellow) in the MOB’s cell layers from female and male voles are shown in Figures 3A–I,J–R, respectively. In the GlL of the MOB, statistically significant differences were found between female groups in the number of BrdU-positive cells (*H* = 10.53, *2df*, *p* = 0.0005; Figure 4A). Similarly, statistically significant differences were found in the number of BrdU/NeuN-positive cells (*H* = 11.28, *2df*, *p* < 0.0001; Figure 4C). Dunn’s *post-hoc* test demonstrates that the number of BrdU-positive cells and new mature neurons increased in the SCM group compared to the C females (*p* = 0.006 and *p* = 0.005; respectively).

In male voles, statistically significant differences were observed in the number of BrdU- (*H* = 11.20, *2df*, *p* = 0.0003; Figure 4A) and BrdU/NeuN-positive cells (*H* = 11.59, *2df*, *p* = 0.0001; Figure 4C) between experimental groups. The Control group expressed a higher number of BrdU-positive cells in contrast to SE and SCM animals (*p* = 0.013 and *p* = 0.019; respectively), whereas C males had more new mature neurons than SE ones (*p* = 0.003; Figures 4A,C) according to *post-hoc* analysis.

No significant differences in the percentage of BrdU/NeuN-positive cells were found between female groups (*H* = 4.74, *2df, p* = 0.089; Table 1), whereas significant differences were observed in male voles (*H* = 6.28, *2df, p* = 0.037; Table 1). SCM voles expressed a higher percentage of new mature neurons than SE animals (*p* = 0.037; Table 1).

**TABLE 1:** Percentage of new mature neurons that survive in the olfactory bulbs and dentate gyrus of the hippocampus.

Region	Cell layer/Sub-region	Females			Males		
		C	SE	SCM	C	SE	SCM
MOB	Glomerular	40.7 ± 3.5	47.6 ± 9.4	56 ± 4.9	53.9 ± 4.6	44.2 ± 2.6 *	61.7 ± 4
	Granular	62.8 ± 1.5	58.1 ± 6.8	53.1 ± 5.2	68 ± 5.7	59.8 ± 4.6	59.2 ± 5.6
AOB	Anterior Glomerular	93.9 ± 3.9	66.7 ± 23.6	61.1 ± 20	44.5 ± 15.9^a^	41.7 ± 25	60 ± 24.5
	Posterior Glomerular	66.7 ± 21.1	25 ± 25	33.3 ± 21.1	33.3 ± 21.1	50 ± 28.9	40 ± 24.5
	Anterior Granular	83.3 ± 16.7	100 ± 0	75.7 ± 16	83.3 ± 16.9	70 ± 23.8	80 ± 20
	Posterior Granular	83.3 ± 16.7	50 ± 28.9	83.3 ± 16.7	66.6 ± 21.1	75 ± 25	60 ± 24.5
DG	Suprapyramidal	54.2 ± 11	65.4 ± 4.6	59.6 ± 8.7	46.7 ± 10.7	63.5 ± 2.5	58.7 ± 3.2
	Infrapyramidal	62.3 ± 7.8	63.2 ± 7.2	70 ± 6.2	57.2 ± 10.2	41.9 ± 8.5	49.6 ± 6

Percentage of new (BrdU) mature neurons (NeuN) in the main (MOB) and accessory (AOB) olfactory bulb and dentate gyrus of the hippocampus in control (C), social exposure (SE), and social cohabitation with mating (SCM) male and female voles. *Different from C. ^a^Different from females in the same group. a different from female p < 0.05.

Sexual dimorphism was observed in the number of both BrdU- and BrdU/NeuN-positive cells between experimental groups. C males had a higher number of both cell populations than C females (*U* = 0, *p* = 0.006 in both conditions); whereas in SCM males these cell populations decreased in comparison to SCM females (*U* = 0, *p* = 0.003 and *U* = 2.5, *p* = 0.011; Figures 4A,C).

#### Granular cell layer

In the GrL from female groups, statistically significant differences were observed in the number of BrdU- (*H* = 14.12, *2df, p* < 0.0001; Figure 4B) and BrdU/NeuN-positive cells (*H* = 13.23, *2df, p* < 0.0001; Figure 4D). Dunn’s *post-hoc* analysis showed that SCM females exhibited a higher number of new cells and new mature neurons than the SE group (*p* = 0.0009; Figures 4B,D).

Male voles did not show significant differences between groups in the GrL from the MOB in the number of BrdU- (*H* = 0.28, *2df, p* = 0.87; Figure 4B) or BrdU/NeuN-positive cells (*H* = 1.77, *2df, p* = 0.44; Figure 4D).

There were no significant differences in the percentage of BrdU/NeuN-positive cells between female (*H* = 2.88, *2df, p* = 0.24; Table 1) or male voles (*H* = 1.55, *2df, p* = 0.49; Table 1).

SCM groups showed sexual dimorphism in the number of BrdU- and BrdU/NeuN-positive cells (*U* = 0, *p* = 0.003 and *U* = 2, *p* = 0.01; respectively. Figures 4B,D), as males exhibited a significant decrease in the number of these cell populations in comparison to females. On the other hand, SE animals presented statistically significant differences both in the number of BrdU-positive cells and in the new mature neurons (*U* = 3, *p* = 0.048 and *U* = 0, *p* = 0.008, respectively; Figure 4D), as males had more cells than females.

### Accessory olfactory bulb

**FIGURE 5:**
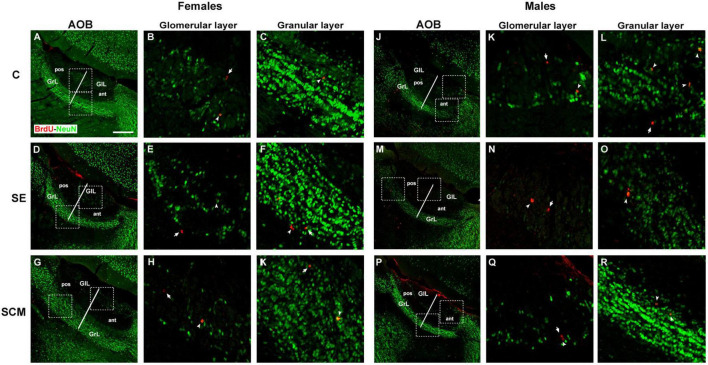
Representative photomicrographs of new neurons that arrive and survive in the accessory olfactory bulb (AOB) after mating and social exposure in female and male prairie voles Photomicrographs of double immunolabeling for BrdU (red; new cells) and NeuN (green; mature neurons) in the glomerular and granular cell layers. **(A–C)** Control (C), **(D–F)** social exposure (SE), and **(G–I)** social cohabitation with mating (SCM) females; **(J–L)** C; **(M–O)** SE and **(P–R)** SCM males. Scattered square: enlargement of the inset at 20×. White arrowheads: BrdU/NeuN-positive cells. White arrows: non-neuron BrdU-positive cells localized in the glomerular and granular cell layers (GlL and GrL, respectively). ant, anterior; pos, posterior. 20×. Scale bar: 100 μm.

Representative photomicrographs of the BrdU- (red) and BrdU/NeuN-positive cells (yellow) in the GrL and GlL of the AOB from female and male voles are shown in Figures 5A–K,J–R, respectively.

#### Anterior glomerular cell layer

**FIGURE 6:**
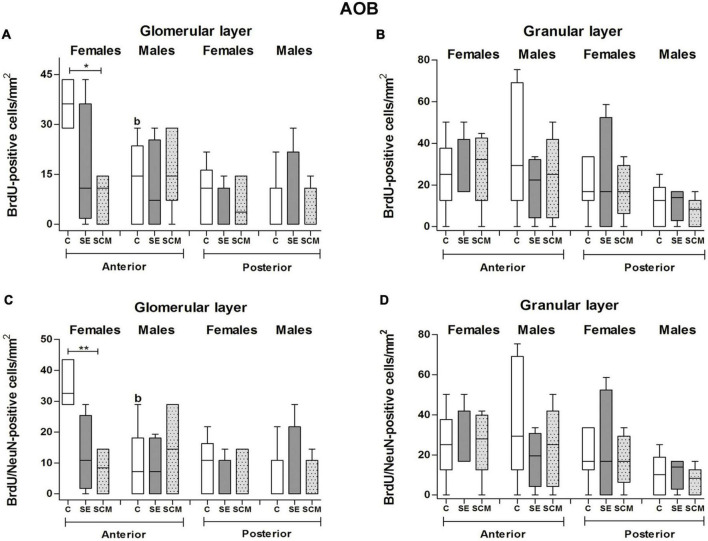
Data analysis of the number of BrdU and BrdU/NeuN-positive cells in the glomerular and granular cell layers of the accessory olfactory bulb (AOB) in control (C), social exposure to a conspecific (SE), and social cohabitation with mating (SCM) groups Kruskal-Wallis followed by Dunn’s *post-hoc* tests; *p < 0.05 and **p < 0.01 were considered significant. Differences between females and males were analyzed using the Mann-Whitney *U* test where ^a^*p* < 0.05, and ^b^*p* < 0.01. Female subjects; C = 6, SE = 4, SCM = 6. Male subjects; C = 6, SE = 4, SCM = 5. ^a^ mean different from the opposite sex *p* < 0.05. ^b^ mean different from the opposite sex *p* < 0.01.

Statistically significant differences between experimental groups were found in female voles both in the number of BrdU- (*H* = 8.39, *2df, p* = 0.008; Figure 6A) and BrdU/NeuN-positive cells (*H* = 10.06, *2df, p* = 0.001; Figure 6C). Control females have more BrdU-positive cells and new mature neurons than SCM animals (*p* = 0.014 and *p* = 0.008, respectively; Figures 6A,C) according to Dunn’s *post-hoc* test. Data analysis from the percentage of BrdU/NeuN-positive cells did not show statistically significant differences between female groups (*H* = 1.44, *2df, p* = 0.52; Table 1).

No statistically significant differences were found in the male vole groups in the number of BrdU- (*H* = 0.69, *2df, p* = 0.73; Figure 6A), BrdU/NeuN-positive cells (*H* = 0.43, *2df, p* = 0.83; Figure 6C) or in the percentage of BrdU/NeuN-positive cells (*H* = 0.61, *2df, p* = 0.75; Table 1).

Sexual dimorphism was observed, as C females showed a higher number of BrdU- and BrdU/NeuN-positive cells and percentage of BrdU/NeuN cells compared with C males (*U* = *1*, *p* = 0.007 and *U* = *1.5*, *p* = 0.009, *U* = 4, *p* = 0.02, respectively; Figures 6A,C and Table 1).

#### Posterior glomerular cell layer

In female voles, no statistically significant differences were found in the number of BrdU- (*H* = 2.99, *2df, p* = 0.73; Figure 6A) and BrdU/NeuN-positive cells (*H* = 3.08, *2df*, *p* = 0.25; Figure 6C).

In male groups, BrdU- (*H* = 1.08, *2df, p* = 0.59; Figure 6A) and BrdU/NeuN-positive cell populations (*H* = 1.08, *2df, p* = 0.60; Figure 6C) exhibited no statistically significant differences.

We did not find statistically significant differences in the percentage of BrdU/NeuN-positive cells between female groups (*H* = 1.99, *p* = 0.55; Table 1) or between male voles (*H* = 0.26, *2df, p* = 0.43; Table 1). Also, no sexual differences were found in this neuronal region.

#### Granular cell layer

##### Anterior granular cell layer

Data analysis from female voles demonstrated no statistically significant differences between groups in the number of BrdU- and BrdU/NeuN-positive cells (*H* = 0.20, *2df, p* = 0.90 and *H* = 0.12, *2df, p* = 0.94, respectively; Figures 6B,D) located in the anterior granular cell region. In male voles, no significant differences were found between experimental groups in the number of BrdU and BrdU/NeuN-positive cells (*H* = 0.74, *2df, p* = 0.71 and *H* = 1.10, *2df, p* = 0.60, respectively; Figures 6B,D).

There were not statistically significant differences in the percentage of BrdU/NeuN-positive cells between female groups (*H* = 2.88, *2df, p* = 0.24; Table 1) or between male groups (*H* = 1.04, *2df, p* = 0.59; Table 1). Nor were differences found between males and females.

##### Posterior granular cell layer

In female voles, data analysis demonstrated no statistically significant differences between groups in the number of BrdU- or BrdU/NeuN-positive cells (*H* = 0.12, *2df, p* = 0.94; Figures 6B,D). In male voles, no significant differences were found between experimental groups in the number of BrdU and BrdU/NeuN-positive cells (*H* = 1.06, *2df, p* = 0.60 and *H* = 0.83, *2df, p* = 0.70, respectively; Figures 6B,D).

No statistically significant differences were found in the percentage of BrdU/NeuN-positive cells (*H* = 1.67, *2df, p* = 0.60 and *H* = 0.38, *2df, p* = 0.88, respectively; Table 1) in female and male voles.

### Dentate gyrus of the hippocampus

#### Suprapyramidal blade

**FIGURE 7:**
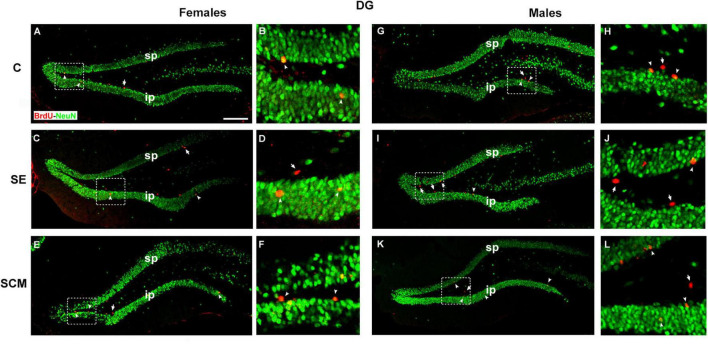
Photomicrographs of double immunolabeling for BrdU (red; new cells) and NeuN (green; mature neurons) in the suprapyramidal (sp) and infrapyramidal (ip) blades **(A,B)**, control (C); **(C,D)** social exposure (SE) and **(E,F)** social cohabitation with mating (SCM) females. **(G,H)** C; **(I,J)** SE and **(K,L)** SCM males. Scattered square: enlargement of the inset at 20×. White arrowheads: BrdU/NeuN-positive cells. White arrows: non-neuron BrdU-positive cells. 20×. Scale bar: 100 μm.

Representative photomicrographs of the BrdU- (red) and BrdU/NeuN-positive cells (yellow) in the DG’s GrL from female and male voles are shown in Figures 7A–F,7G–L, respectively.

**FIGURE 8:**
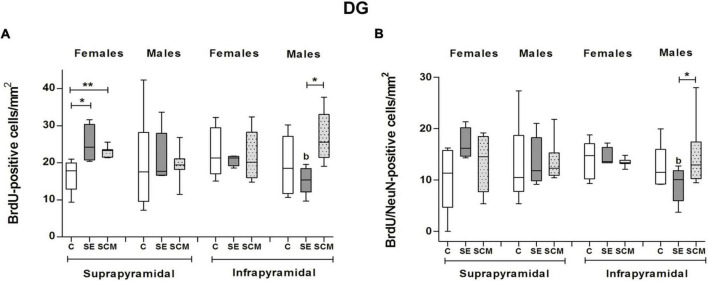
Data analysis of the number of BrdU and BrdU/NeuN-positive cells in the suprapyramidal and infrapyramidal blades of the DG for Control (C), Social exposure to a conspecific (SE) and Social cohabitation with mating (SCM) female and male prairie voles Kruskal-Wallis followed by Dunn’s *post-hoc* tests; *p < 0.05 and **p < 0.01 were considered significant. Differences between females and males were analyzed using the Mann-Whitney *U* test, *^a^p* < 0.05 and *^b^p* < 0.01. Female subjects; C = 7, SE = 4, SCM = 7. Male subjects; C = 6, SE = 5, SCM = 7.

In the suprapyramidal blade of the DG from female voles, statistically significant differences were observed in the number of BrdU-positive cells (*H* = 11.56, *2df, p* = 0.0004; Figure 8A) between experimental groups. Dunn’s *post-hoc* analysis showed that SE and SCM females exhibited a higher number of new cells than C voles (*p* = 0.02 and *p* = 0.08, respectively; Figure 8A). No significant differences were found in the number of BrdU/NeuN-positive cells (Figure 8B).

Male groups did not show significant differences in the number of BrdU- or BrdU/NeuN-positive cells (*H* = 0.30, *2df, p* = 0.87; Figures 8A, H = 2.67, *2df, p* = 0.27; Figure 8B, respectively). No significant differences were found between male and female voles.

We did not find statistically significant differences in the percentage of BrdU/NeuN-positive cells between female and male animals (*H* = 0.11, *2df, p* = 0.95 and *H* = 2.27, *2df, p* = 0.34; respectively, Table 1).

#### Infrapyramidal blade

Data analysis from the infrapyramidal blade in female voles did not show statistically significant differences in the number of BrdU-positive cells and BrdU/NeuN-positive cells (*H* = 0.11, *2df, p* = 0.95; Figure 8A and *H* = 3.02, *2df, p* = 0.23; Figure 8B).

In male groups, statistically significant differences were observed in the number of BrdU- and BrdU/NeuN-positive cells (*H* = 7.78, *2df*, *p* = 0.013; Figure 8A, and *H* = 8, *2df, p* = 0.01, Figure 8B); whereas *post-hoc* analysis using Dunn’s test show that SCM animals expressed a higher number of new cells and new neurons as compared with SE voles (*p* = 0.016; Figure 8A, and *p* = 0.02; Figure 8B).

Data analysis of females vs. males demonstrated that SE male voles expressed a fewer number of BrdU- (*U* = 0, *p* = 0.016; Figure 8A) and BrdU/NeuN-positive cells (*U* = 0, *p* = 0.016; Figure 8B) than SE females.

There were no significant differences in the percentage of BrdU/NeuN-positive cells between female and male groups (*H* = 1.10, *2df, p* = 0.58 and *H* = 0.21, *2df, p* = 0.60; respectively, Table 1).

## Discussion

### Effects of social exposure and social cohabitation with mating in the survival of new cells and neurons in the main and accessory olfactory bulb

#### Females

In the present study, SCM increases the number of new cells and newly generated neurons that survive in the GlL of the MOB of female voles (Figures 4A,C and Table 2). The GlL is formed by two principal interneuron subtypes (i.e., dopaminergic and calretinin cells), and a limited number of calbindin glutamatergic interneurons (Lledo and Valley, 2016). Indeed, new dopamine and calretinin cells are continually integrated during adult neurogenesis (Ninkovic et al., 2007; Adam and Mizrahi, 2011). Given that dopaminergic cells process odor cues, the reduction of dopamine neurons, receptors or transporters decreases the capability to discriminate odors (Taylor et al., 2009; Paß et al., 2020). On the other hand, calretinin interneurons are responsible for inhibiting noisy excitatory signals that reach mitral/tufted cells (Capsoni et al., 2021). Interestingly, a pool of calretinin periglomerular cells remains in an immature stage for a long time, and it has been proposed that these cells could supply glomerular networks dependent on specific sensory experiences (Benito et al., 2018). Here, we proposed that in SCM females, these new cells that survive in the GlL are likely dopaminergic or calretinin interneurons, which can contribute to the female prairie voles’ ability to discriminate odors. However, further studies are needed to determine if GlL new cells are indeed dopamine or calretinin positive cells in the female prairie vole.

**TABLE 2:** Summarized effects of social exposure and social cohabitation with mating over new cells and neurons that survival in the olfactory bulbs and dentate gyrus of the hippocampus.

Region	Cell layer/Sub-region	Females		Males		Sex dimorphism	
		New cells	New neurons	New cells	New neurons	New cells	New neurons
MOB	Glomerular	↑ SCM (vs. C)	↑ SCM (vs. C)	↓ SE, SCM (vs. C)	↓ SE (vs. C)	C ♂ >♀	C ♂ >♀
						SCM ♂<♀	SCM ♂ >♀
	Granular	↑ SCM (vs. SE)	↑ SCM (vs. SE)	ns	ns	SE ♂ >♀	SE ♂ >♀
						SCM ♂<♀	SCM ♂<♀
AOB	Anterior Glomerular	↓ SCM (vs. C)	↓ SCM (vs. C)	ns	ns	C ♂<♀	C ♂<♀
DG	Suprapyramidal	↑ SE, SCM (vs. C)	ns	ns	ns	ns	ns
	Infrapyramidal	ns	ns	↑ SCM (vs. SE)	↑ SCM (vs. SE)	SE ♂<♀	SE ♂<♀

Summary of the principal findings in cell survival in control (C), social exposure (SE), social cohabitation with mating (SCM) groups in the main olfactory bulb (MOB), accessory olfactory bulb (AOB), and dentate gyrus (DG) of the hippocampus. ↑ Increases, ↓ decreases; < or > represent significant differences p < 0.05; ns, no statistic differences were found. ♂ male, ♀ female.

In this study, the number of new cells and neurons that survive in the GrL increases in SCM compared to SE female voles in the MOB (Figures 4B,D and Table 2). This difference could be explained by the fact that voles from the SCM group were housed with their sexual partner for 45 days and were constantly exposed to volatile and non-volatile sexually relevant odors, sexual, and social stimuli. In contrast, SE animals were exposed to opposite-sex sensorial cues and volatile sexual relevant odors only for 6 h in a single session (Figure 1).

Granule cells are small inhibitory interneurons and the largest neuronal population in the OB that regulates odor information sent from excitatory projection neurons to the olfactory cortex. Postnatally born neurons are integrated into the deep GrL circuits (Sakamoto et al., 2014). Adult newborn granular cells from the mouse MOB have a higher response to novel odors than mature preexisting neurons, as evaluated by the expression of early genes (Magavi et al., 2005). In female mice, OB adult neurogenesis disruption induced alterations in their interaction with males, but not with females (Feierstein et al., 2010). Feierstein and coworkers, proposed a decrease adult neurogenesis in the OB could induce deficits in the detection or processing of male odors lead to altered gender recognition (Feierstein et al., 2010). Thus, it is possible in the SCM female voles, the new cells that survive in the GlL and GrL of the MOB could be more sensitive to sexual odor cues from their male partner and contribute to their recognition.

Consistently, Smith and collaborators reported that female vole exposure to non-family related males increases cell proliferation in the RMS and enhances the commitment to the neuronal lineage (Smith et al., 2001). Similarly, female rats that mated in 4 or 10 sessions (with behavioral tests applied every 5 days) showed an increase in the neuronal lineage differentiation in the MOB (Alvarado-Martínez and Paredes, 2018; Portillo et al., 2019). Moreover, exposure to male odors increases newborn cells in the MOB (Mak et al., 2007; Larsen et al., 2008).

In the anterior AOB, the SCM condition decreases the number of new cells and new periglomerular neurons in the GlL in comparison to control animals (Figures 6A,C and Table 2). No statistically significant differences between groups were found in the posterior GlL of the AOB and in the anterior and posterior GrL. These results show that sexual behavior and cohabitation with a male decreases cell survival in the anterior GlL of the AOB in female voles and suggest that a different neural plastic mechanism could be involved in the process of social cohabitation with mating information to this region.

In contrast with our findings reported here, in non-monogamous rodent species the first sexual stimulation increases the percentage of new surviving neurons in the GrL of the AOB in female rats (Corona et al., 2016), whereas repeated mating increases cell survival in the GlL and the percentage of new neurons in the GlL and GrL of the AOB (Alvarado-Martínez and Paredes, 2018; Portillo et al., 2019). In female mice, exposure for 7 days to male odors increases newborn cells in the pAOB (Nunez-Parra et al., 2011), whereas exposure to male-soiled bedding, but not to its volatile odors only, increases the number of new neurons in the anterior and posterior AOB of female mice (Oboti et al., 2009).

#### Males

Data from the present study showed SCM and SE groups presented a decrease in the number of new surviving cells in the GlL of the MOB; whereas in this layer the SE group showed decreases the number of new surviving neurons in comparison to the control group (Figures 4A,C). No statistically significant difference was found between groups in the GrL of the MOB (Figures 4B,D and Table 2). Similarly, no statistically significant differences in the number of new cells and new neurons were found in the anterior and posterior GlL and GrL of the AOB. Our previous findings demonstrated that SCM and SE for 6 h did not increase cell proliferation in the SVZ and migration in the RMS in male voles. However, SCM and SE increase new neuroblast percentages in the dorsal region of the SVZ compared with the control group (Castro et al., 2020). Thus, in agreement with our proliferation studies (Castro et al., 2020), socio-sexual stimulation did not modify cell survival in the GrL of the MOB and AOB in male voles.

Surprisingly, in this study, in male voles SE and SCM decreased cell survival and SE reduced neuronal differentiation in the GlL of the MOB, whereas SCM decreased the number of newborn cells and neurons in the GlL of the anterior AOB in females. Indeed, adult neurogenesis can decrease in response to aversive social experiences, such as acute and chronic stress, depression-like behaviors, interaction with dominant and aggressive conspecifics, and social isolation (Gould et al., 1997; Mirescu and Gould, 2006; Lieberwirth et al., 2012). Other socio-sexual behaviors, such as motherhood, reduce cell proliferation and survival in the OB and DG in California mice, rats, and sheep (Leuner et al., 2007; Brus et al., 2010, 2014; Glasper et al., 2011). Fatherhood also decreases cell survival in the DG of monogamous California mice (Glasper et al., 2011) and prairie voles (Lieberwirth et al., 2013). In prairie voles, fatherhood decreases cell survival in the DG, hilus and molecular cell layer, but not in the granular layer. No effects were observed in the MOB (Lieberwirth et al., 2013). Decrease in hippocampal cell survival could be explained by sexual and pair bonding experiences since pup exposure or circulating corticosterone did not decrease cell survival. In contrast, sexual behavior that leads to pair bonding and fatherhood increases anxiety and depression-like behaviors in prairie voles, which can lead to reduced adult neurogenesis (Lieberwirth et al., 2013). Consistently, Rochefort and Lledo (2005) reported an increase in new bulbar neurons when animals were housed in an enriched environment, but these populations returned to control levels a month later in adult mice. These data could explain the decrease in the number of newborn survival cells in the GlL of the MOB in SCM male voles.

Cell death and adult neurogenesis are necessary for new memory formation. Weisz and Argibay (2009, 2012), using computational simulations of hippocampal function, proposed that adult neurogenesis expands the hippocampus’s capacity for new information while high levels of hippocampal neurogenesis increasing the forgetting of old information, as these are transferred to the neocortex for long-term storage. Continuous integration of new neurons may affect previous memories stored in the preexisting circuits (Frankland, 2013). Accordingly, inhibition of neurogenesis in mice prevents forgetting of the hippocampus-dependent memories (Akers et al., 2014). Thus, the new neurons facilitate recent memory processing and promote the gradual replacement of old memories, which can be transfer to the cerebral cortex for permanent storage, allowing the hippocampus available to process new events (Feng et al., 2001).

To our knowledge, there are no studies determining whether new neurons in the OB can contribute to the storage of old olfactory memories to another brain regions. It is possible the decrease in cell survival induced by socio-sexual stimulation in the GlL of the MOB in males and the GlL of the aAOB in female voles can be another mechanism involved in the olfactory memory signature of the sexual partner. Further studies are needed to determine the physiological relevance of reduced bulbar neurogenesis in prairie voles.

### Effects of social exposure and social cohabitation with mating in the survival of new cells and neurons in the dentate gyrus of the hippocampus

#### Females

Our findings reveal that neural plasticity of the DG of adult female and male voles differentially responds to sociosexual behavior. For example, in females, SCM and SE conditions increase the number of new surviving cells with respect to control animals in the suprapyramidal blade, but no effects were observed over neuronal differentiation (Figures 8A,B and Table 2). Other authors previously reported that new hippocampal neurons had heightened synaptic plasticity, high input resistance and a lack of GABAergic inhibition leading to hyperexcitability and lower activation thresholds as compared with mature neurons, also presenting increased calcium conductance and a lower threshold for inducing long-term potentiation in comparison to mature hippocampal neurons (Schmidt-Hieber et al., 2004; Ge et al., 2007; Toni et al., 2008; Gu et al., 2012; Drew et al., 2016). Therefore, it has been proposed that new mature neurons can participate in new memory formation and regulate the activity of mature granule cells (Schmidt-Hieber et al., 2004; Gu et al., 2012).

Our research group and others have reported an analogous situation, where the first sexual experience increases the number of new cells in the ventral blade, while mating in four sessions also increases the number of new cells in the dorsal and ventral blade in female rats (Portillo et al., 2012). However, Fowler and collaborators demonstrated that cohabitation with mating or cohabitation with a female did not increase cell proliferation or survival in the DG, neither increase the percentage of new cells that differentiate into neurons in female prairie voles (Fowler et al., 2002). These differences may be due to the fact that Fowler and coworkers used gonadally intact females and for their survival studies and females that cohabitated with the male were sacrificed 3 days following litter birth, so pregnancy and parturition can also influence adult neurogenesis (Pawluski et al., 2022).

### Males

Here, we reported that the SCM group had more new cells and new mature neurons than SE voles in the infrapyramidal blade of the DG (Figures 8A,B and Table 2). We previously reported that SCM and SE increase the proliferation of new cells in the whole DG, including the hilus, whereas in the infrapyramidal blade, SCM and SE to another male increase the number of newly generated neuroblasts in male prairie voles (Castro et al., 2020). Consistent with, in male rats one sexual activity increments cell proliferation in the DG of the hippocampus, and repeated sexual intercourse for 14 consecutive days increases cell proliferation and survival in the DG (Leuner et al., 2010). Middle-age male rats that mate for 14 or 28 days had an increase in neurogenesis in the DG (Glasper and Gould, 2013). Contradictory results were reported by Spritzer and coworkers, who reported that male rats that mate in four mating tests with a novel sexual partner in each test, neurogenesis decreases in the DG. Phenomena were not observed in males that mate repeatedly with the same female and in the control group (Spritzer et al., 2016). Thus, mating could increase neurogenesis in the DG and the differences in surviving cells between SCM and SE vole males could be explained due to sexual stimulation and not only the sensorial cues from the females. Also, they lived with an opposite sex animal for 45 more days. Interestingly, the increase in the number of new cells and neurons was exclusively in the infrapyramidal blade, a region involved in emotional behavior, social interactions, social recognition, mnemonic processing of odor and reward information and stress resilience (Kesner, 2018). The ventral DG of the hippocampus also is important in reward memory retrieval of contextual cues (Kesner, 2018). In male voles, cohabitation with mating induces a reward state mediated by opioids, however, social exposure without mating is not rewarding (Ulloa et al., 2018). Rivera and coworkers demonstrated in male mice, that adult born DG cells are involved in the maintenance of a recently formed opioid-associate reward memory (Rivera et al., 2019). Thus, voles from the SCM group, sexual behavior will increase new cells/neurons survival in the ventral DG and new cells could be involved in sexual partner rewarding memory.

Previous studies in rats showed that both blades of the DG exhibit a subtle asymmetry in anatomic and physiological properties. These differences are accentuated in epileptic rats who express a greater mossy fiber sprouting in the infrapyramidal blade, presenting hyperexcitability in granule neurons (Scharfman et al., 2002). These results suggest that the suprapyramidal and infrapyramidal blades of the DG could have different circuit functions and connections. Consistently, granule cells from these sub-regions express heterogeneity in their electrophysiological properties such as excitability, action potential and frequency-dependent response (Chawla et al., 2005).

Further, in mice and rats, different dendritic morphology has been observed between granule cells from both blades. Granular cells from the suprapyramidal blade express more dendrites in the region that receives spatial information from the medial perforant pathway (Gallitano et al., 2016). These antecedents and the results reported in the present study demonstrate that sociosexual behavior could modulate cell proliferation, survival, and integration to preexisting neuronal circuits in both supra- and infrapyramidal blades of the DG, and these new neurons would be favoring spatial memory and social and sexual/partner recognition in adult prairie voles (Lieberwirth et al., 2016).

### Sexual dimorphism in adult cell survival

In the present study, sex differences were found in the GlL of the MOB; control males had more new cells and new neurons than control females. But no differences between control males and females were found in the GrL of the MOB (Figure 4 and Table 2). Since SCM decreases the survival of new cells in male voles, female voles from the SCM group exhibited a higher number of new cells and new mature interneurons in the GlL than males (Figures 4A,C and Table 2). Similarly, SCM female voles had a higher number of new cells and new granule neurons than SCM males in the GrL of the MOB (Figures 4B,D and Table 2). In the SE condition, males had more new cells and mature new granular interneurons as compared with females (Figures 4B,D and Table 2). In the anterior AOB, control females had more new cells and newly generated neurons than control males in the GlL (Figure 6C and Table 2).

Our data disagree with mouse studies in which 2-month-old males had a higher number of new cells in the AOB than females. Interestingly, this dimorphism was not maintained in older animals, where no differences were found between males and females in the MOB (Nunez-Parra et al., 2011). Oboti and coworkers did not find differences in adult neurogenesis between sexes in the GrL of the AOB and MOB in mice (Oboti et al., 2009). Another study reported that adult male rats have more new GrL cells in the anterior AOB than females, but no sex differences were found in the MOB (Peretto et al., 2001).

Here, in prairie voles, SE females expressed more new cells and mature neurons than males in the infrapyramidal blade of the DG (Figures 8A,B). Consistently, neurospheres derived from the DG were larger in control female voles as compared with males, suggesting that female neural stem cells had an enhanced proliferative potential (Ávila-González et al., 2020).

## Conclusion

Our study suggests than social and sexual stimulation and cohabitation with a partner modulates new cells/neurons survival in a region-specific and sex dependent manner in the prairie vole, a social monogamous specie (Table 2). In female voles, social cohabitation with mating increases the number of new cells and neurons in the GlL (vs. control group) and GrL (vs. social exposure group) of the MOB and the number of new cells that survive in the suprapyramidal blade of the DG (vs. control group). These new cells/neurons may be involved in sexual partner memory formation. Unexpectedly, in males, social cohabitation with mating decreases the number of new cells and neurons that survive in the GlL of the MOB (vs. control group) and in females decrease the number of new cells/neurons in the GlL of the aAOB (vs. control group). The physiological relevance of this reduction in cell survival needs further investigation. In the DG of the hippocampus of females, social exposure and social cohabitation with mating increase the new cells that survive in the suprapyramidal blade, region involved in spatial memory. For male voles, social cohabitation with mating increases the new cells and neurons than survive in the infrapyramidal blade of the DG, region involved in reward value and social recognition. Our study also demonstrates that cell survival in the OB and DG is sexually dimorphic, region-specific, and dependent of the socio-sexual stimulation. Further studies are needed to elucidate the role of the newly generated cells and new interneurons in response to sociosexual experience in the prairie voles. We hypothesize that these cells/neurons could be integrated into the preexisting olfactory or hippocampal network and modulate partner recognition and memory, consolidating pair bonds and social interactions with opposite-sex conspecifics.

## Data availability statement

The raw data supporting the conclusions of this article will be made available by the authors, without undue reservation.

## Ethics statement

The animal study was reviewed and approved by the Animal Care Committee of the Instituto de Neurobiologia (072) and Ethical Committee of the Instituto Nacional de Perinatologia (Registry number: 2018-1-163).

## Author contributions

AC performed the experiments, analyzed the data, and contribute to the writing. RD-O performed the experiments and analyzed the data. LY and RP acquired funding, reviewed, and edited the manuscript. FC and DÁ-G performed the experiments. ND and WP conceptualized the study, acquired funding, and contribute to the writing. All authors contributed to the article and approved the submitted version.

## Footnotes

1
www.graphpad.com
